# A point mutation resulting in a 13 bp deletion in the coding sequence of *Cldf* leads to a GA-deficient dwarf phenotype in watermelon

**DOI:** 10.1038/s41438-019-0213-8

**Published:** 2019-12-01

**Authors:** Chunhua Wei, Chunyu Zhu, Liping Yang, Wei Zhao, Rongxue Ma, Hao Li, Yong Zhang, Jianxiang Ma, Jianqiang Yang, Xian Zhang

**Affiliations:** State Key Laboratory of Crop Stress Biology in Arid Areas, College of Horticulture, Northwest A&F University, Yangling, Shaanxi 712100 China

**Keywords:** Agricultural genetics, Genetic markers

## Abstract

The dwarf architecture is an important and valuable agronomic trait in watermelon breeding and has the potential to increase fruit yield and reduce labor cost in crop cultivation. However, the molecular basis for dwarfism in watermelon remains largely unknown. In this study, a recessive dwarf allele (designated as *Cldf* (*Citrullus lanatus dwarfism*)) was fine mapped in a 32.88 kb region on chromosome 09 using F_2_ segregation populations derived from reciprocal crossing of a normal line M08 and a dwarf line N21. Gene annotation of the corresponding region revealed that the *Cla015407* gene encoding a gibberellin 3β-hydroxylase functions as the best possible candidate gene for *Cldf*. Sequence analysis showed that the fourth polymorphism site (a G to A point mutation) at the 3′ AG splice receptor site of the intron leads to a 13 bp deletion in the coding sequence of *Cldf* in dwarf line N21 and thus results in a truncated protein lacking the conserved domain for binding 2-oxoglutarate. In addition, the dwarf phenotype of *Cldf* could be rescued by exogenous GA_3_ application. Phylogenetic analysis suggested that the small multigene family *GA3ox* (GA3 oxidase) in cucurbit species may originate from three ancient lineages in Cucurbitaceae. All these data support the conclusion that *Cldf* is a GA-deficient mutant, which together with the cosegregated marker can be used for breeding new dwarf cultivars.

## Introduction

Dwarfism is a valuable and economically important plant architecture trait in crop breeding, and has positive effects on improving yield and high efficiency in labor reduction in management and harvesting strategies. Various dwarf mutants discovered in different plant species have been widely used in crop breeding, such as the Green Revolution genes *sd1* in rice and *Rht-D1b* and *Rht-B1b* in wheat^1,2^. In cucurbits, dwarf or compact plant types have attracted much attention from plant breeders because of the higher planting densities to improve crop production. To date, several recessive genes conferring short internodes or bushy phenotypes have been reported in cucumber. For example, the truncated F-box protein CsaVBF1 is strongly associated with dwarfism in cucumber mutant *si*^3^. A CLAVATA1-type receptor-like protein, *CsCLAVATA1*, in cucumber was considered the best possible causal gene for the dwarf phenotype in the EMS-induced mutagenesis *Csdw*^4^. Additionally, a putative cytokinin oxidase gene *CKX* identified in the *cp* locus seemed to be responsible for the compact habit in cucumber line PI308915^5^, while the BR-C6 oxidase-encoding gene *CsCYP85A1* and steroid 5α-reductase encoding gene *CsDET2* from cucumber dwarf mutants *scp-1* and *scp-2*, respectively, were confirmed to be functionally involved in brassinosteroid (BR) biosynthesis^6,7^. Compact growth habits in melon, including short internode and short lateral branching, are regulated by recessive or incomplete dominant genes, such as *si-1*, *si-2*, *si-3*, *mdw1*, and *slb*, which have not yet been cloned^8–10^. In squash, bushy plant habit is a dominant phenotype and controlled by the *Bu* locus, which also lacks functional characterization^11^.

Genes underlying the dwarf mutations were mainly involved in biosynthesis or the signal transduction pathway of plant hormones, such as gibberellins (GAs)^1,2^, cytokinin^5^, and BRs^6,7^, which regulate cell elongation and division. GAs, a class of important plant growth-promoting hormones, have been reported to play critical roles in controlling plant growth and development^1,12,13^. Diverse mutations in the biosynthetic and metabolic pathways of GAs producing bush types enable the elucidation of the underlying genetic basis of dwarfism. For example, Green Revolution genes *sd1* in rice and *Rht-D1b* and *Rht-B1b* in wheat were reported to be involved in GA metabolism and signaling response pathways, which encode nonfunctional GA20 oxidase (GA20ox) and DELLA proteins without functional DELLA domains, respectively^1,2^. As the hub repressors in the GA signaling transduction pathway, DELLA proteins belonging to the GRAS gene family contain both N-terminal DELLA and VHYNP domains^13,14^. In wheat, the aforementioned *Rht-D1b* and *Rht-B1b* mutations in the N-terminal motif lead to reduced responsiveness to GA and dwarfism^2^. Additionally, the deletion of the 17 amino acid residue segment in the DELLA domain of GAI reduces plant height in *Arabidopsis*^15^. In the GA biosynthetic pathway, the CPS encoding enzyme is involved in an early step, which converts the GGDP to CDP in plastids, while KAO in the endoplasmic reticulum catalyzes the conversion of *ent*-kaurene acid GA_12_^12,16,17^. GA3 oxidase (GA3ox), as well as GA2 oxidase (GA2ox) and GA20ox, is important for the production of biologically active GAs in the final steps^13,18^. In monocots, mutations in *GA3ox* (GA 3β-hydroxylase), such as *Dwarf1* (*D1*) from maize and *OsGA3ox1* and *OsGA3ox2* (*Dwarf18* or *D18*) from rice, exhibit dwarfism^19,20^. To date, several GA 3β-hydroxylase genes have also been characterized in dicot species^21–24^. The *GA4* gene encoding a 3β-hydroxylase was reported to be involved in GA biosynthesis in *Arabidopsis*^21^. The *GA4*-related protein Le with GA 3β-hydroxylation activity is able to convert GA_20_ to bioactive GA_1_, and its mutant allele *le* leads to a dwarf phenotype in pea^22^. Moreover, some *GA3ox* genes have also been functionally characterized in cucurbit crops, such as watermelon, cucumber, and pumpkin^25–27^. Notably, unlike the DELLA GA signaling mutants, the dwarf phenotype of GA biosynthetic mutants can be rescued, in some cases, by the application of exogenous GAs^28^.

Watermelon (*Citrullus lanatus* L.) is an economically important cucurbit crop, which accounts for 7% of the vegetable production area worldwide^29^. In watermelon, four genes conferring dwarfism have been reported, including gene *dw-1* and its allele *dw-1*^*s*^, and two independent loci *dw-2* and *dw-3*^30–33^. Recently, a recessive locus named *dsh* has been located on chromosome 7, and the gene *Cla010726* encoding a GA20ox-like protein is recognized as the most possible candidate gene^34,35^. In this study, we fine mapped a new dwarf locus, *Cldf* (*Citrullus lanatus dwarfism*), and gene *Cla015407*, encoding a GA 3β-hydroxylase, was recognized as the best possible causal gene. Sequence analysis revealed that the fourth polymorphism site (a G to A transition) at the 3′ AG splice receptor site of the intron leads to a 13 bp deletion in the coding sequence of *Cldf* in dwarf line N21 and thus results in a truncated protein lacking the conserved domain for binding of 2-oxoglutarate. Examination of exogenous GA_3_ application confirmed that *Cldf* is a GA-deficient mutant. Phylogenetic analysis suggested that there may be three ancient *GA3ox* lineages in the common ancestor of Cucurbitaceae. This new dwarf mutant line as well as the cosegregated marker will be helpful for breeding new watermelon cultivars with a dwarfism phenotype.

## Materials and methods

### Plant materials and morphological characterization

Two watermelon inbred lines used as parents in this study, M08 and N21, were grown in a greenhouse on the campus of Northwest A&F University, Yangling, China. M08 is an ordinary inbred material with normal vines, while line N21 with short internodes shows a dwarfism phenotype. For inheritance analysis and causal gene identification, two distinct F_1_ generations (N21 × M08 F_1_ and M08 × N21 F_1_) were generated by bidirectional crossing with two parental lines N21 and M08. Then, ten plants for each F_1_ generation were self-pollinated and individually harvested to produce F_2_ segregating populations. Subsequently, seven M08 × N21 F_2_ populations with a total of 1474 plants and three N21 × M08 F_2_ populations with 618 individuals were used for linkage analysis and identification of candidate genes for *Cldf*. Germinated seeds of two parental lines, as well as the F_1_ and F_2_ progenies, were directly sown in plastic pots and transferred to greenhouses under natural conditions at the third-leaf stage.

The phenotypes were visually recorded twice at seedling and mature stages and then classified as dwarfism or normal. The deviation from the expected 3:1 segregation ratio in the F_2_ population was tested using the *χ*^2^ test. To investigate the plant height of parental lines and F_1_ progeny, five individuals for each generation were randomly selected and measured with an ordinary steel ruler. The length of 22 internodes for each plant was also recorded. Using the SPSS 21.0 software, Duncan’s test was used to evaluate the significance of statistical data.

### Whole-genome re-sequencing of two parental lines

Genomic DNA from young leaves of two parental lines was extracted using the CTAB (cetyl trimethylammonium bromide) method^36^. The quality of extracted genomic DNA was examined on a 1% agarose gel, and the purity was checked by a Nanodrop2000 spectrophotometer (Thermo Scientific, Wilmington, DE). Using the Illumina HiSeq X Ten platform, the genomes of two parental lines were re-sequenced to generate 150 bp paired-end reads by BioMarker Co. (Beijing, China).

### Data analysis and marker development

After removing the adaptors, reads with more than 10% unknown bases, and low-quality reads, the clean data were mapped onto the reference genome of watermelon 97103 (http://www.icugi.org/) using the BWA software^37^. Raw single-nucleotide polymorphism (SNP) and indel calling was carried out via SAMtools software^38^. Then, after discarding the low-quality SNPs with read depths <20, high-confident SNPs and indels were obtained and used to develop corresponding CAPS (cleaved amplified polymorphic sequence) markers with the Primer Premier 5 software (http://www.premierbiosoft.com/). The sequencing data are accessible in the NCBI database under accession numbers SAMN11080422 and SAMN11080423. To validate the genomic polymorphism sites, all the reads mapped on candidate genes were visually investigated and compared between two parental lines using the JBrowse software^39^.

### Molecular mapping of the *Cldf* locus

To preliminarily locate the *Cldf* locus, we designed one polymorphic marker for each chromosome based on the high-confidence SNPs identified above. Then, these 11 markers were used to screen a small F_2_ segregation population with 96 individuals. After initial chromosome anchoring of the *Cldf* locus, new flanking markers were developed to genotype the small population. Subsequent to delimiting the dwarf locus to a primary mapping interval, a larger population was used to identify recombinants. Then, four new polymorphic markers in the primary mapping region were designed and used to screen the recombinants to narrow down the mapping interval. Primer information of all the polymorphic markers is listed in Supplementary Table S1.

### Candidate gene prediction and pathway-related gene identification

The annotated genes in the final mapping interval were analyzed according to the reference genome 97103. The genomic and coding sequences of the candidate gene were independently amplified from M08, N21, and 97103 and were then sent for sequencing. The software Geneious (http://www.geneious.com) was used to perform sequence analysis.

To date, the GA biosynthesis and signaling transduction pathways have been well characterized, in which genes encoding different functional enzymes have been cloned^13,16,18^. We retrieved amino acid sequences of one *CPS1* (*At4g02780*) gene, two *KAOs* (*KAO1*, *At1g05160*; *KAO2*, *At2g32440*), five *GA20oxs* (*AtGA20ox1*, *At4g25420*; *AtGA20ox2*, *At5g51810*; *AtGA20ox3*, *At5g07200*; *AtGA20ox4*, *At1g60980*; *AtGA20ox5*, *At1g44090*), three *GID1s* (*GID1a*, *At3g05120*; *GID1b*, *At3g63010*; *GID1c*, *At5g27320*), and five *DELLAs* (*RGA*, *At2g01570*; *GAI*, *At1g14920*; *RGL1*, *At1g66350*; *RGL2*, *At3g03450*; *RGL3*, *At5g17490*) from *Arabidopsis* via the TAIR database (www.arabidopsis.org). Using protein sequences as queries, the respective homologs were identified in watermelon via the Blastp program (*E* value setting of 1.0 × 10^−5^).

### RNA extraction and qRT-PCR analysis

For tissue-specific analysis, the roots, leaves, stems, tendrils, and both male and female flowers were independently sampled from lines M08 and N21. To analyze the expression levels of pathway-related genes, two adjacent unexpanded internodes from apical shoots were also independently harvested from two parental lines.

Using the RNA Simple Total RNA Kit (Tiangen, China), total RNA was extracted from the harvested samples, and the first strand complementary DNA (cDNA) was synthesized via the FastKing RT Kit with gDNase (Tiangen, China). Amplification was performed in a 20 µL reaction volume containing 10.0 µL of SYBR Green Premix (TaKaRa), 1.0 µL of cDNA template (80 ng/µL), 0.8 µL of each primer (10 µM), and 7.4 µL of ddH_2_O. Using a StepOnePlus Real-Time PCR System (Applied Biosystems, Foster, USA), the PCR amplification conditions included pre-denaturation for 5 min at 95 °C, followed by 40 cycles of 95 °C for 10 s and 60 °C for 30 s. The housekeeping gene *Cla007792* was used as an internal reference^40^, and the relative expression level for each gene (three biological and three technical replicates) was calculated using the 2^−∆∆Ct^ method^41^. All gene-specific primers used in quantitative reverse transcription PCR (qRT-PCR) experiments are listed in Supplementary Table S1.

### Measurement of endogenous GA_3_ application

Homozygous recessive individuals at the four-leave stage were selected from the F_2_ population and used to treat endogenous GA_3_. GA_3_ powder was first dissolved in a small amount of ethanol and then diluted with ddH_2_O to the final concentration (200 mg/L). Seedlings sprayed with an equal volume of the corresponding mixture (ethanol and ddH_2_O) without GA_3_ were used as a control. For each treatment, nine seedlings were chosen and sprayed with endogenous GA_3_ or the corresponding mixture at 4-day intervals six times. Then, the plant height was measured with ordinary tapeline. After removing two maximum and two minimum values, the statistical data (five for each treatment) were analyzed with Student’s *t* test to evaluate the significance.

### *GA3ox* homolog identification and phylogenetic analysis

For genome-wide identification of *GA3oxs* in watermelon, the amino acid sequence of the *Cla015407* gene was used as a query to blast against the predicted protein file (v1, reference genome 97103) using the Blastp program (*E* value cutoff of 1.0 × 10^−10^). The reliability of candidate ClGA3oxs was validated through searching against the TAIR and NCBI databases. Then, using the reliable *ClGA3ox* candidates as queries, *GA3ox* homologs were genome widely identified from cucumber (*Cucumis sativus*, v3), melon (*C. melo*, v4), pumpkin (*C. maxima*, v1.1), and bottle gourd (*L. siceraria*, v1). All the predicted protein files of cucurbit species were downloaded from CuGenDB (http://cucurbitgenomics.org/). The amino acid sequences of published GA3oxs in different species, such as watermelon (*ClGA3ox1*)^27^, cucumber (*CsGA3ox1* to *CsGA3ox4*)^25^, pumpkin (*CmGA3ox1* to *CmGA3ox4*)^26^, tomato (*SlGA3ox1* and *SlGA3ox2*)^42^, grape (*VvGA3ox1* to *VvGA3ox3*)^43^, *Arabidopsis* (*AtGA3ox1* to *AtGA3ox4*)^44^, soybean (*GmGA3ox1* to *GmGA3ox6*)^45^, maize (*ZmGA3ox1* and *ZmGA3ox2*)^20^, and rice (*OsGA3ox1* and *OsGA3ox2*)^19^, were retrieved according their GenBank accession number or gene ID (Supplementary Table S2).

Multiple sequence alignment of full-length proteins was constructed via the Muscle software^46^. A neighbor-joining tree was generated with 1000 bootstrap replicates using MEGA 6.0^47^.

## Results

### Phenotypic characterization and inheritance of the dwarfism trait

**Fig. 1:**
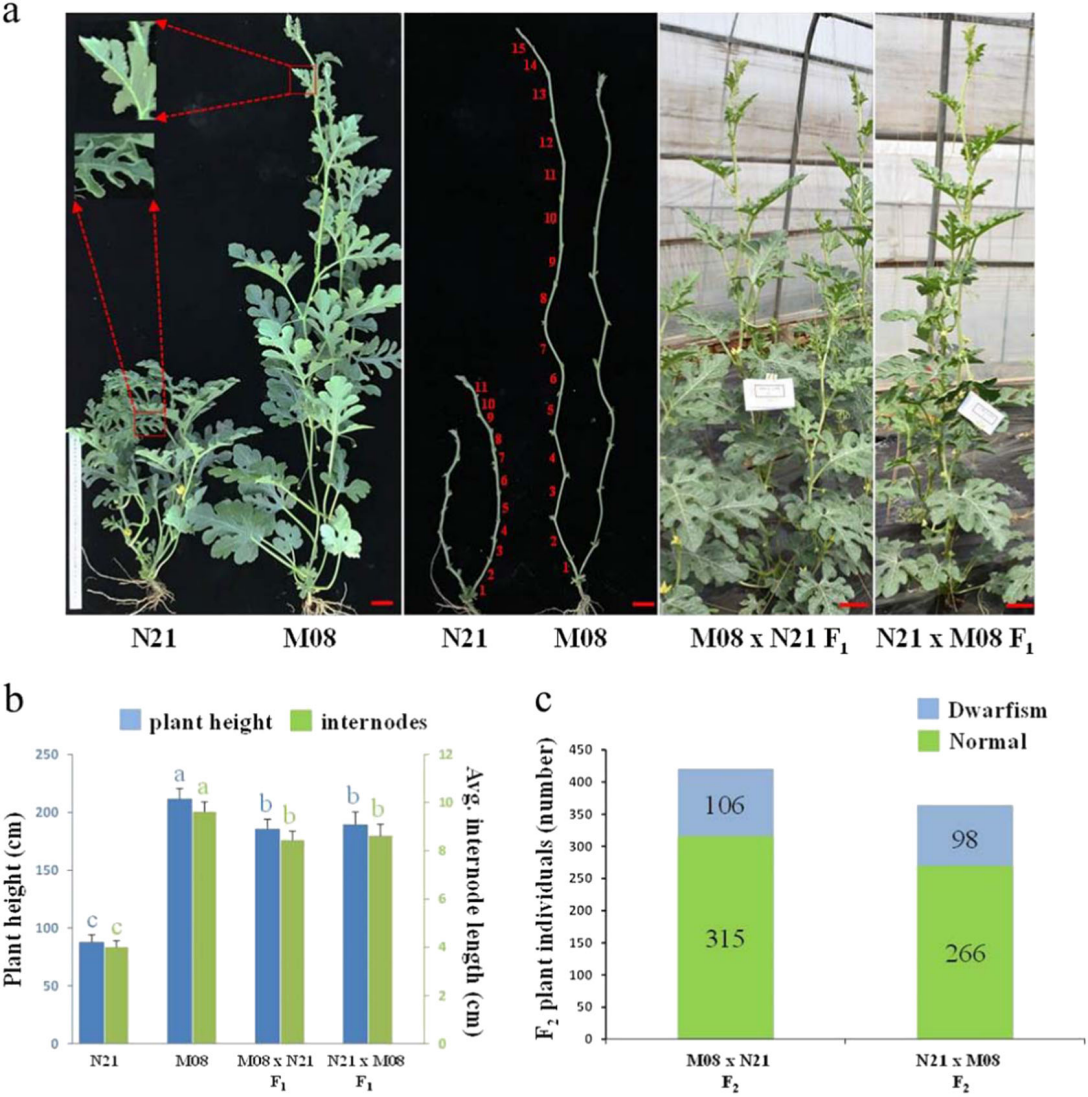
Phenotypic characterization and statistical data analysis of watermelon dwarf line N21, normal line M08, and reciprocal crossing of F_1_ hybrids. **a** Morphological characterization of four watermelon lines. Magnified views of unexpanded leaves in two parental lines show different morphological types of leaf margins. The numbers in red represent internodes in the main stems. Bar = 5 cm. **b** The plant height and internode length of four watermelon lines. The blue and green bars represent plant height (five individuals for each line) and average internode length (22 internodes for each individual) data, respectively. The data are presented as the mean ± SD. Duncan’s test was conducted for statistical analysis. Different letters refer to significance at *p* < 0.05. **c** The number of dwarf and normal phenotype individuals in reciprocally crossed F_2_ segregation populations.

Compared with the normal line M08, the dwarf inbred line N21 with smaller leaves showed compact plant architecture (Fig. 1a). The objective phenotype can be visibly distinguished at the seedling stage (Supplementary Fig. S1a) and obviously classified as dwarfism or normal throughout the whole development stage. In addition, other morphological traits are also different between the two lines, such as leaf size, shape index, and trichome density of ovaries, and petals of male flowers (Supplementary Fig. S1b–d). Notably, the margin of the young leaf was slightly curled in line N21, and its growth vigor was much weaker than M08 (Fig. 1a). To compare the internode length and plant height among two parents and their F_1_ progenies, five individuals for each line were randomly selected. The internode length of N21 (4.0 ± 0.8 cm, 22 internodes) was much shorter than that of M08 (9.6 ± 1.7 cm, 22 internodes), and the plant height of the former (88.0 ± 6.1 cm) was also significantly less than that of the latter (211.6 ± 8.9 cm) (Fig. 1b). The plant height of reciprocally crossed F_1_ plants, as well as internode length, was also significantly higher than the N21 dwarf line, but significantly less than the ordinary line M08, indicating that the normal vine phenotype is dominant to dwarfism.

To analyze the inheritance of the dwarfism phenotype, we collected phenotypic data from two reciprocal F_2_ segregation populations. As shown in Fig. 1c, there were 315 normal and 106 dwarf plants in the M08 × N21 F_2_ population (total 421 individuals), fitting a 3:1 Mendelian ratio (*χ*^2^ = 0.0008, p = 0.93). Moreover, 364 N21 × M08 F_2_ individuals contained 266 normal and 98 dwarf vine plants, which was also consistent with the Mendelian ratio of 3:1 (*χ*^2^ = 0.62, p = 0.40). Taken together, these data suggest that the dwarfism phenotype in N21 is controlled by a single recessive gene and is hereafter designated *Cldf*.

### Genome-wide identification of high-confidence SNPs and indels

To obtain enough SNPs and indels for developing polymorphic marks, genomes of two parental lines were re-sequenced. After removing low-quality reads, we obtained a total of 39.0 and 36.6 million clean reads for lines M08 and N21, respectively, with ~11.7 and 11.0 GB of data and Q30 values above 93.0%, respectively (Table 1). Then, 98.59% and 98.25% of these clean reads for M08 and N21, respectively, could be successfully mapped on the reference genome, resulting in a total of 936,540 SNPs and 165,962 indels between two genomes. After discarding the low-quality sites with read counts <20, 152,894 high-confidence SNPs and 4018 indels were obtained and utilized to develop CAPS markers in the mapping strategy (Supplementary Table S3).

**Table 1:** Detailed characteristics of the DNA-seq data of M08 and N21.

	M08	N21
Number of clean reads	39,016,840	36,630,657
Clean data (bp)	11,686,884,478	10,967,051,578
Q30 percentage	93.08%	93.44%
GC%	35.11%	35.12%
Mapped reads	38,466,703 (98.59%)	35,989,621 (98.25%)
Coverage ratio	96.60%	94.50%

### Linkage mapping of the dwarfism locus *Cldf*

**Fig. 2:**
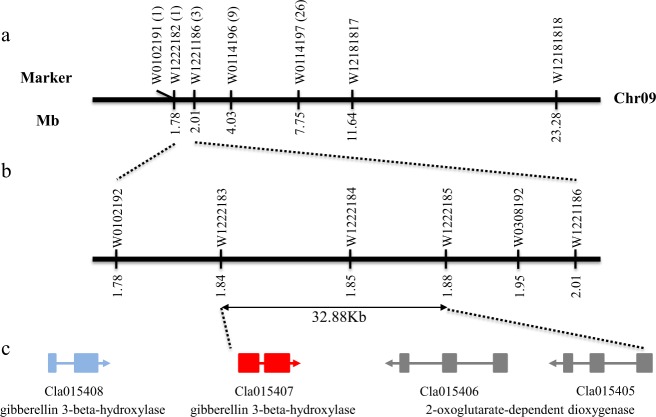
Map-based cloning of the dwarfism gene *Cldf*. **a** Primary mapping of *Cldf*. The *Cldf* gene was preliminarily located between markers W1222182 and W1221186 on chromosome 09. The numbers within brackets indicate the number of recombinants between markers and phenotypes. **b** Fine mapping of the *Cldf* gene. Gene *Cldf* was finally delimited in a 32.88 kb region between markers W1222183 and W1221185. Marker W1221184 cosegregates with the phenotypes. **c** Schematic diagram of predicted genes. Three genes were annotated in the mapping interval, and the *GA3ox* homolog *Cla015407* (in red) encoding a gibberellin 3β-hydroxylase was considered the most possible candidate gene. Another flanking *GA3ox* homolog outside the mapping region, *Cla015408*, was marked in blue.

A recent study reported that the *Cla010726* gene on chromosome 7 encoding a GA20ox-like protein functions as the most possible candidate gene in watermelon mutant *dsh*^35^. To validate whether *Cla010726* is the causal gene in line N21, a nearby polymorphic marker W12181814 was designed to screen a small M08 × N21 F_2_ population (96 individuals: 70 normal and 26 dwarf plants, *p* = 0.64 in *χ*^2^ test against 3:1 segregation ratio). Linkage analysis indicated that marker W12181814 was not linked with the dwarfism trait, inferring that the underlying gene *Cldf* in N21 is not *Cla010726*. To locate the *Cldf* gene on the chromosome, polymorphic markers were designed for the other ten chromosomes (data not shown) and then used to genotype individuals in the small population mentioned above. As a result, the marker W12181817 on chromosome 9 was confirmed to be linked with the dwarfism locus (Fig. 2a). Then, another three polymorphic markers (W0102191, W0114197, and W12181818) were designed to screen this small population. Subsequent linkage analysis implied that the dwarfism locus was delimited to a 5.97 Mb genomic region between markers W0102191 and W0114197, with 1 and 26 recombinants, respectively. Then, to narrow down this mapping interval, three new polymorphic markers (W1222182, W1221186, and W0114196) were designed to screen the 27 recombinants. Finally, the dwarfism trait was delimited within a 235.67 kb region between markers W1222182 and W1221186 (Fig. 2a), with 1 (1.04 cM) and 3 (3.13 cM) recombinants, respectively.

To further narrow down the initial mapping interval, the remaining larger segregating populations, including 325 M08 × N21 F_2_ and 364 N21 × M08 F_2_ individuals, were subjected to genotype with the primary flanking markers W1222182 and W1221186. Another eight new recombinants were identified from the M08 × N21 F_2_ generation, while only one recombinant was from the F_2_ offspring of N21 × M08. A total of 13 recombinants were used for further mapping of the dwarfism locus. Four new polymorphic markers (W1222183, W1222184, W1222185, and W0308192) were developed to genotype the 13 recombinants (Fig. 2b). Finally, the *Cldf gene* was delimited between markers W1222183 and W0308192, with one and seven recombinants, respectively. Two markers, W1221184 and W1221185, cosegregated with the phenotype. The physical distance of the fine mapping region was 106.72 kb, with two boundary markers W1222183 and W0308192 at 0.24 and 1.66 cM away from the dwarf locus, respectively.

To further precisely map the *Cldf* gene, additional individuals, including 1053 from the M08 × N21 F_2_ population and 254 from N21 × M08 F_2_, were genotyped with the above primary flanking markers W01222182 and W1221186. An additional 17 recombinants were obtained, including 15 from the M08 × N21 F_2_ population and 2 from N21 × M08 F_2_, which were also subjected to genotype with the four markers (W1222183, W1222184, W1222185, and W0308192). As a result, the *Cldf* locus was finally narrowed down to a 32.88 kb region between markers W1222183 and W1222185, with two recombinants for each marker (Fig. 2b). One cosegregated marker, W1221184, was obtained, which can be used for marker selection breeding programs.

### Identification of candidate genes for the dwarfism gene *Cldf*

According to the annotated version of the reference genome, only three genes were annotated in the fine mapping region (Fig. 2c). Two homologous genes, *Cla015405* and *Cla015406*, encode 2-oxoglutarate-dependent dioxygenase protein. The third *Cla015407* gene is predicted to encode a GA 3β-hydroxylase (also named GA3ox), which is predicted to be involved in the final step of GA biosynthesis^13,16,18^. It is worth noting that another GA 3β-hydroxylase coding gene, *Cla015408*, is located outside the mapping interval, sharing 83.28% amino acid similarity with *Cla015407*. According to our re-sequencing data, we first analyzed the genomic polymorphisms of these four genes between two parents (Supplementary Fig. S2). As a result, no polymorphic sites were found in three genes (*Cla015408*, *Cla015405*, and *Cla015406*), while four SNP mutations and one indel mutation were identified in *Cla015407* between the two parental lines. Hence, we proposed that the *Cla015407* gene is the most likely causal gene underlying the dwarfism phenotype in the N21 line.

**Fig. 3:**
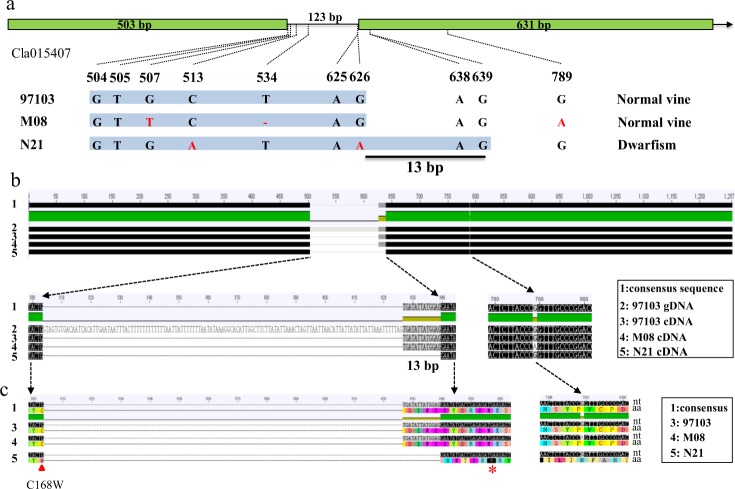
Sequence analysis of candidate gene *Cla015407* among N21, M08, and reference genotype 97103. **a** Schematic diagram of genomic variations of *Cla015407* among three genotypes. The physical positions of four SNPs and one indel between two parental lines are presented. The third SNP (G to A) in line N21 is predicted to lead to a 13 bp deletion in the coding sequence of *Cldf*. **b** Sequence alignment of the coding sequence of *Cla015407* among the three genotypes. The 13 bp deletion was confirmed in the cDNA in line N21. **c** Alignment of predicted amino acid sequences of *Cla015407* among three genotypes. The translation frameshift started at the 168 residue (C to W). A premature stop codon in *Cldf* resulted in a truncated protein with only 173 amino acid (aa) residues. The synonymous mutation in the exon with no aa conversion is also represented.

According to the genome annotation, the total nucleotide length of *Cla015407* is 1257 bp and contains two exons (503 and 631 bp) and a 123 bp intron (Fig. 3a). To confirm the genomic variations observed above, we cloned the genomic sequence of this candidate gene from N21 and M08 and then compared them with the reference sequence from genome 97103, which is an East Asia watermelon cultivar with normal vines^29^. Undoubtedly, a total of five SNPs/indels were obtained, with four in the intron and one existing in the second exon (Fig. 3a). The first three polymorphisms in the intron, as well as that in the exon, were predicted with no effect on the gene structure or amino acid sequence changes.

Numerous studies have confirmed that introns probably possess a dinucleotide GT (splice donor site) at the 5′ boundary and an AG dinucleotide pair (splice receptor site) at the 3′ end^48^. The fourth mutation in the intron (G to A in line N21) may affect the original splicing of the intron and result in a 13 bp deletion in CDS of *Cldf* (Fig. 3a). To validate this assumption, we cloned the coding sequences of *ClDF* and *Cldf* alleles from two parents and the reference 97103 genotype. Sequence alignment showed that the cDNA sequences of *ClDF* in both M08 and 97103 are 1134 bp long and predicted to encode 377 amino acid residues, while a 13 bp deletion was found in *Cldf* occurring exactly at the fourth point mutation mentioned above (Fig. 3b). Moreover, this deletion could lead to frameshift translation and a premature stop codon, producing a truncated protein with only 173 amino acid residues (Fig. 3c). It is worth noting that the premature stop codon caused the lack of the conserved motif NyYPXCXXP (Supplementary Fig. S3) in Cldf, which is involved in the binding of 2-oxoglutarate^43^. Hence, we inferred that the fourth SNP in the intron is the causal mutation, which changes the function of *Cldf* in the dwarf line.

### Expression analysis of *ClDF*/*Cldf* alleles and pathway-related genes

**Fig. 4:**
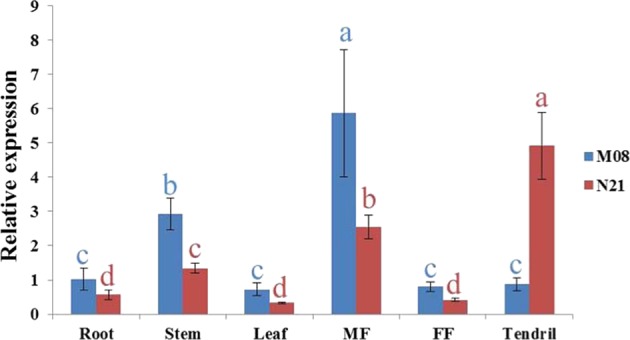
Expression analysis of *ClDF and Cldf* in different tissues of two parental lines. The transcriptional level of the respective gene in roots (M08) was set to a value of 1 and used as a reference. The data are shown as the mean ± SD. Different letters refer to significance at *p* < 0.05 (Duncan’s test). FF = female flowers; MF = male flowers.

We examined the transcript abundance of *Cla015407* by qRT-PCR in roots, leaves, stems, tendrils, and male and female flowers (Fig. 4). Compared with roots, the expression of *ClDF* in M08 was upregulated in stems and male flowers, with the highest transcript accumulation in male flowers. In dwarf line N21, the mutant allele *Cldf* was increased in stems, male flowers, and tendrils, of which the latter showed the highest transcriptional abundance. Similar expression patterns were observed in the five organs between two parental lines, except for tissue tendrils (Fig. 4).

**Fig. 5:**
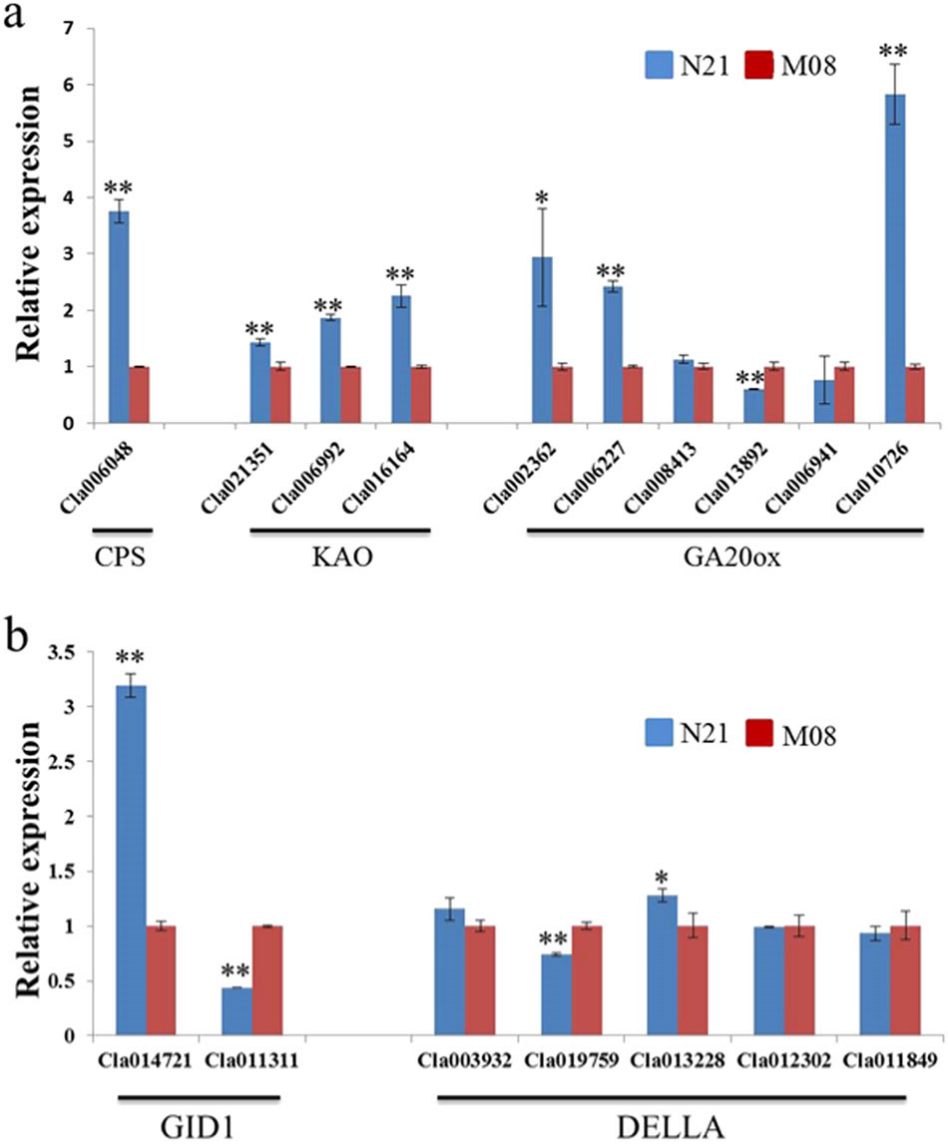
Expression analysis of GA biosynthesis (**a**) and signaling transduction (**b**) pathways related genes in the stems of two parental lines. The transcriptional level of the respective genes in stems (M08) was set to a value of 1 and used as a reference. The data are shown as the mean ± SD. *^,^** represent significant differences in expression levels at *p* < 0.05 and *p* < 0.01, respectively (Student’s *t* test).

To date, the GA biosynthesis and signaling transduction pathways have been well characterized, and genes encoding different functional enzymes at each step in the pathways have been cloned (Supplementary Fig. S4)^13,16,18^. Using the amino acid sequences of the *CPS1* gene, two *KAOs* (*KAO1* and *KAO2*) and five *GA20oxs* (*AtGA20ox1*, *AtGA20ox2*, *AtGA20ox3*, *AtGA20ox4*, and *AtGA20ox5*) from *Arabidopsis* as queries^45,49,50^, we identified one *CPS1* homolog (*Cla006048*), three *KAO* homologs (*Cla021351*, *Cla006992*, and *Cla016164*), and five *GA20ox* homologs (*Cla002362*, *Cla006227*, *Cla008413*, *Cla013892*, and *Cla006941*) in watermelon genome 97103 (Supplementary Table S4). The expression level of the dwarf candidate gene *Cla010726* was also analyzed, which was published recently and predicted to encode a GA20ox-like protein^35^. Compared with the expression pattern in line M08, all four genes involved in the GA biosynthesis pathway were upregulated in unexpanded internodes of N21 (Fig. 5a). Similarly, three *GA20ox* homologs (*Cla002362*, *Cla006227*, and *Cla010726*) were significantly upregulated in dwarf line N21, while only the *Cla013892* gene was downregulated compared to that in line M08. The GA receptor GID1 that was first identified in rice contains three orthologous copies (AtGID1a, AtGID1b, and AtGID1c) in *Arabidopsis*, which were confirmed with some overlapping but also distinct functions in plant developmental processes^51^. The active GA-GID1 complex could trigger the rapid degradation of DELLA proteins via the 26S proteasome pathway, which act as GA signaling repressors and contain five members (RGA, GAI, RGL1, RGL2, and RGL3) in *Arabidopsis*^16,52–54^. In watermelon, we identified two GID1 (*Cla014721* and *Cla011311*) and five DELLA (*Cla003932*, *Cla019759*, *Cla013228*, *Cla012302*, and *Cla011849*) homologous genes (Supplementary Table S4). Interestingly, the transcription levels of GID1 homolog *Cla014721* and DELLA gene *Cla013228* were induced in dwarf line N21, while the expression of genes *Cla011311* (GID1) and *Cla019759* (DELLA) was significantly reduced in N21 compared to that in normal line M08 (Fig. 5b). Additionally, the transcription levels of the other three DELLA genes were not obviously different between the two parental lines.

### Recovery of dwarfism phenotype by exogenous GA_3_ application

**Fig. 6:**
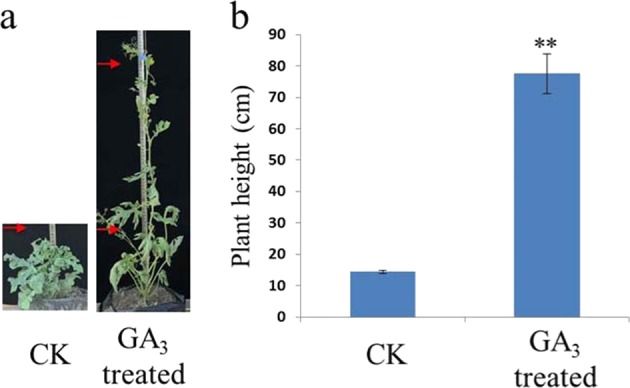
Recovery of the *Cldf* mutant by exogenous GA_3_. **a** Phenotypes of *Cldf* mutant seedlings that were treated with exogenous GA_3_ (200 mg/L). Red arrows indicate 20 cm (bottom) and 70 cm (top). **b** Plant heights of *Cldf* mutant seedlings treated with exogenous GA_3_ (200 mg/L). Plants sprayed with an equal volume of the corresponding mixture without GA_3_ were used as a control in this experiment. Nine seedlings were used for each treatment (details in “Materials and methods”). After removing two maximum and two minimum values, the statistical data (five for each treatment) were analyzed with Student’s *t* test to evaluate the significance. ** indicates a statistically significant difference in expression level at *p* < 0.01.

It has been reported that the dwarf phenotype of GA biosynthetic mutants can be rescued, in some cases, by the application of exogenous GA_3_^4,28^. Therefore, we investigated the phenotypes of homozygous recessive individuals from the F_2_ population, which were treated with exogenous GA_3_ application (200 mg/L, detailed in Materials and methods section). As shown in Fig. 6, plant heights could be rescued by the application of GA_3_. In addition, the leaf color and leaf margin treated with exogenous GA_3_ were also restored to M08 appearance (Supplementary Fig. S1e).

### Phylogenetic analysis of candidate gene *Cldf*

**Fig. 7:**
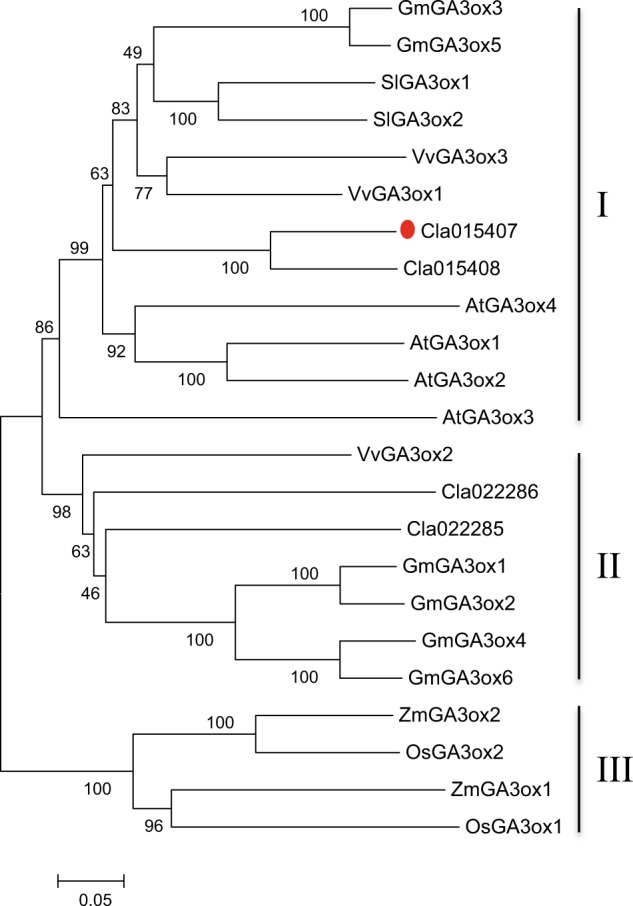
Phylogenetic analysis of the *GA3ox* gene family in watermelon and six other species. Three groups can be found in the distance tree. The candidate gene *Cla015407* is highlighted in red. Numbers on nodes represent bootstrap values.

Increasing evidence has shown that enzymes involved in the final steps of the GA biosynthesis pathway are encoded by small multigene families^19,20,42–44^. Recently, *GA3ox* genes have been identified and cloned from several species, such as tomato, grape, *Arabidopsis*, soybean, maize, and rice^19,20,42–45^. Using *Cla015407* as a query, we identified four *ClGA3ox* homologs in watermelon, which displayed two exons according to the gene annotation (Supplementary Table S5). A phylogenetic tree was constructed with protein sequences of four ClGA3oxs and homologs from tomato (two SlGA3oxs), grape (three VvGA3oxs), *Arabidopsis* (four AtGA3oxs), soybean (six GmGA3oxs), maize (two ZmGA3oxs), and rice (two OsGA3oxs) (Fig. 7). Homologs from monocot species (maize and rice) formed an independent lineage (group III) in the distance tree, while those from dicot genomes could be divided into two groups (I and II), which is similar to the topologies observed in published studies^43,55^. In the dicot lineage, group I contained homologs from five dicotyledon species, including the target gene Cla015407 and its flanking homolog Cla015408, while members in group II were only from three species, inferring their ancient origination in dicot plants.

To further investigate the evolutionary history of the *GA3ox* family in Cucurbitaceae, we identified 24 homologs genome-wide from four other cucurbit species, including *C. sativus*, *C. melo*, *C. maxima*, and *L. siceraria* (Supplementary Table S5). Notably, all the cloned GA3oxs in *C. lanatas*, *C. sativus*, and *C. maxima* were included in our identification^25–27^. Then, a total of 28 GA3oxs from Cucurbitaceae together with 19 homologs from other families were aligned to generate a distance tree, which could also be divided into three groups (Supplementary Fig. S5). Interestingly, members from cucurbit genomes mingled together in group I, while those from other species formed independent clades, which is similar to subgroup IIb. In subgroup IIa, *GA3ox* homologs were only from cucurbit species, inferring that this lineage may be specific to Cucurbitaceae.

## Discussion

Watermelon is an important cucurbit crop worldwide, which accounts for 7% of the global vegetable production area^29^. Plant height in watermelon is a vital agronomic architecture trait that can increase fruit yield and reduce labor costs in crop cultivation and pruning. There are four reported genes conferring dwarfism in watermelon, including gene *dw-1* and its allele *dw-1*^*s*^, as well as independent loci *dw-2* and *dw-3*^30–33^. In a previous study, a new recessive locus named *dsh* was located on chromosome 7, and the *Cla010726* gene encoding a GA20ox-like protein was recognized as the best possible candidate gene^34,35^. Linkage analysis indicated that gene *Cla010726* is not the causal gene in dwarf line N21, although similar morphological traits were observed in both compact materials, such as numerous branches and small and curled leaves (Fig. 1a)^34,35^. Moreover, our work presented herein suggested that a GA 3β-hydroxylase encoding gene *Cla015407* was recognized as the best possible candidate gene leading to the dwarfism phenotype in line N21 (Figs. 2 and 3). Sequence analysis identified four SNPs and one indel in the genomic sequences of *Cla015407* between two parental lines (Fig. 3a and Supplementary Fig. S2). Numerous studies have confirmed that introns probably contain the canonical splice model possessing the consensus 5′ GT splice donor site and the 3′ AG splice receptor site^48^. The fourth polymorphic site (G to A) disrupted the original splicing site of the intron in *Cldf*, resulting in a 13 bp deletion in the coding sequence (Fig. 3b). The mutant allele *Cldf*, which carries a premature stop codon that produces a truncated protein with only 173 amino acid residues, lacks the conserved motif NyYPXCXXP (Supplementary Fig. S3), which is considered to be involved in the binding of 2-oxoglutarate^43^. In addition, the dwarf phenotype could be recovered by the application of exogenous GA_3_ (Fig. 6). Overall, it is reasonable to speculate that the *GA3ox* homolog *Cla015407*, which is involved in the final step of the GA_3_ biosynthesis pathway, is the causal gene for the dwarfism phenotype in watermelon line N21.

The biosynthesis of active GAs is a complex and multistep process that recruits different functional enzymes to catalyze diverse intermediates (Supplementary Fig. S4). Gene CPS functioning in an early step of the GA biosynthetic pathway can convert GGDP to CDP in plastids, while KAO in the endoplasmic reticulum catalyzes the conversion of *ent*-kaurene acid GA_12_^12,16,17^. GA3ox and GA20ox play important roles in the final steps of the GA biosynthesis pathway^13,18^. In our study, the transcription levels of both *CPS* and *KAO* homologs, as well as three *GA20ox* members, were upregulated in dwarf line N21 compared to that in M08 (Fig. 5a), suggesting possible feedback regulation. Consistent with our conclusion, the levels of *GA3ox* and *GA20ox* transcripts increased in the *kao1 kao2* double mutant of *Arabidopsis*^49^. The dynamic balance of active GAs in plants is maintained by DELLA-dependent feedback regulation of GA biosynthesis genes^12^. The active GA-GID1 complex could trigger rapid degradation of the master GA signaling repressor DELLA proteins^16^, while increasing DELLA activity obviously results in the accumulation of *GA3ox1* and *GA20ox1* transcripts^49,56^. The expression of *GA*_*4*_ (*AtGA3ox1*) was reduced 26% by GA_3_ treatment in the *Arabidopsis rga* (DELLA) mutant^56^. In the present study, five DELLA homolog genes were identified in watermelon (Supplementary Table S4), of which only one gene, *Cla019759*, was repressed in dwarf mutant *Cldf* (Fig. 5b). As the GA receptor, three GID1 orthologous copies (GID1A, GID1B, and GID1C) were confirmed to have overlapping but also functional specificity in regulating different developmental processes^51,57^. Moreover, GA treatment resulted in feedback inhibition of all three *AtGID1* genes^57^. In this study, expression analyses revealed that the two *ClGID1s* exhibited distinct expression patterns between two parental lines (Fig. 5b), suggesting their possible distinct functions.

Numerous studies have shown that enzymes involved in the final steps of the GA biosynthesis pathway are encoded by small multigene families^19,20,42–44^. In plants, GA3oxs convert GA_12_ to bioactive GAs in the final step of the biosynthesis pathway^13,16,18^. In *Arabidopsis*, four *GA3ox* homologs have been identified and designated *AtGA3ox1* to *AtGA3ox4*, which exhibit organ-specific expression patterns and some degree of functional redundancy^44^. Similarly, two *GA3ox* members in rice, *OsGA3ox1* and *OsGA3ox2*, also showed different expression patterns^19^. In cucumber and pumpkin, four *GA3ox* genes have been characterized in each genome with possible redundant and specific functions^25,26,55^. In addition, a previous study reported that gene *Cv3h* (a *GA3ox* homolog and identified as *Cla022286* in this study) may function in the developing seeds of watermelon^27^. Here, we infer that another *GA3ox* homologous gene, *Cla015407*, is responsible for internode elongation in dwarf line N21. Moreover, the germination rate of N21 seeds is much lower than that of M08 (data not shown), inferring that these two *ClGA3oxs* may have overlapping and specific functions. To further recover the evolution of the *GA3ox* family in plants, a phylogenetic tree was constructed with 28 GA3oxs from Cucurbitaceae and 19 homologs from other families. As shown in Supplementary Fig. S5, homologs prefer to gather together at the family level in different groups/subgroups, which is consistent with the observations in previous studies^43,45,55^. Additionally, it seems that there are three ancient lineages in the common ancestor of Cucurbitaceae, and one of them (subgroup IIa) is specific to cucurbit crops.

## Supplementary information

Table S1

Table S2

Table S3

Table S4

Table S5

Fig S1

Fig S2

Fig S3

Fig S4

Fig S5
