# Supplementary figures and images for: A point mutation resulting in a 13 bp deletion in the coding sequence of Cldf leads to a GA-deficient dwarf phenotype in watermelon
Source: Hortic Res. 2019 Dec 1;6:132. doi: 10.1038/s41438-019-0213-8 (PMC6885051; doi:10.1038/s41438-019-0213-8)

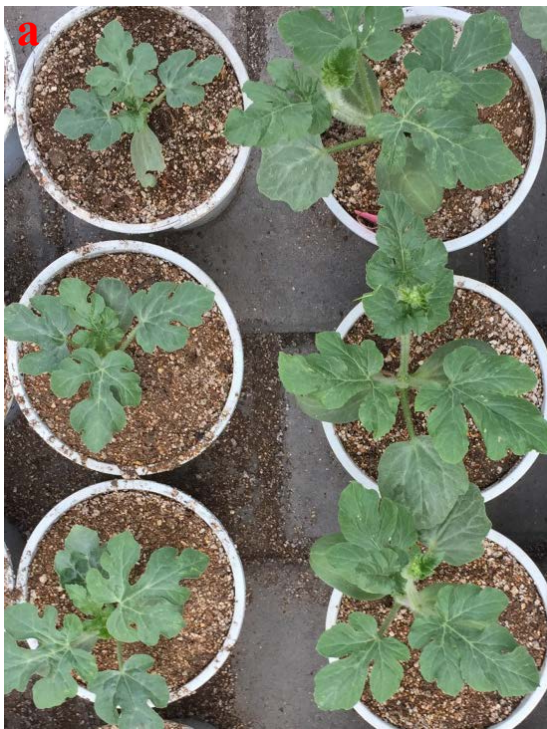

**N21**

**M08**

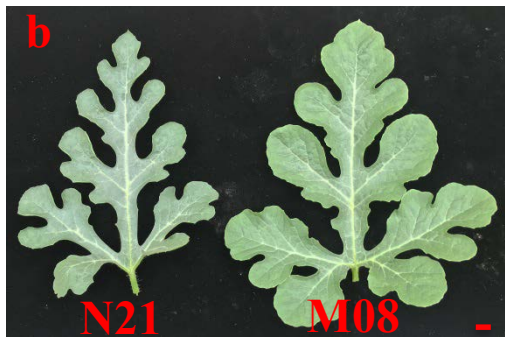

**N21**

**M08**

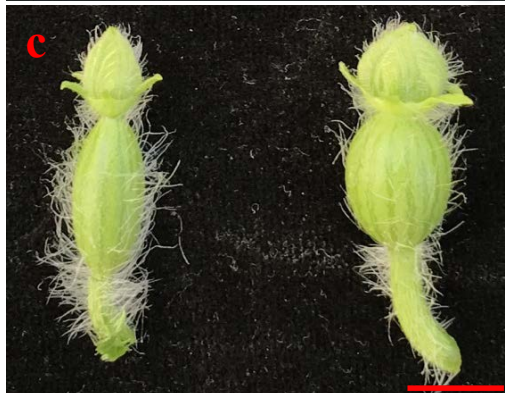

**N21**

**M08**

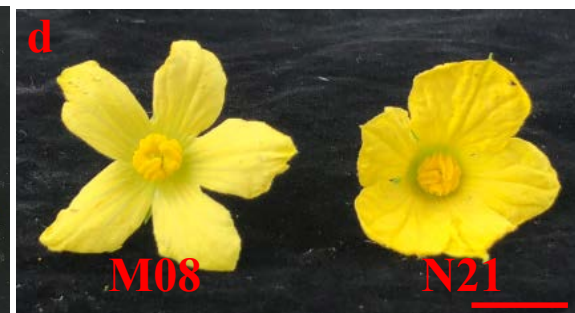

**M08**

**N21**

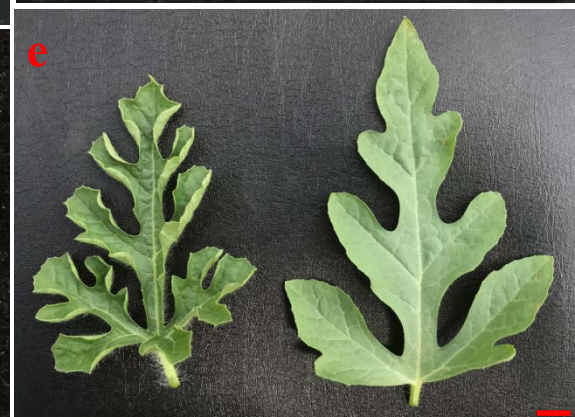

**e**

Supplement: Supplementary file 6 — Fig S1 [file 41438_2019_213_MOESM6_ESM.pdf]

Cla015408

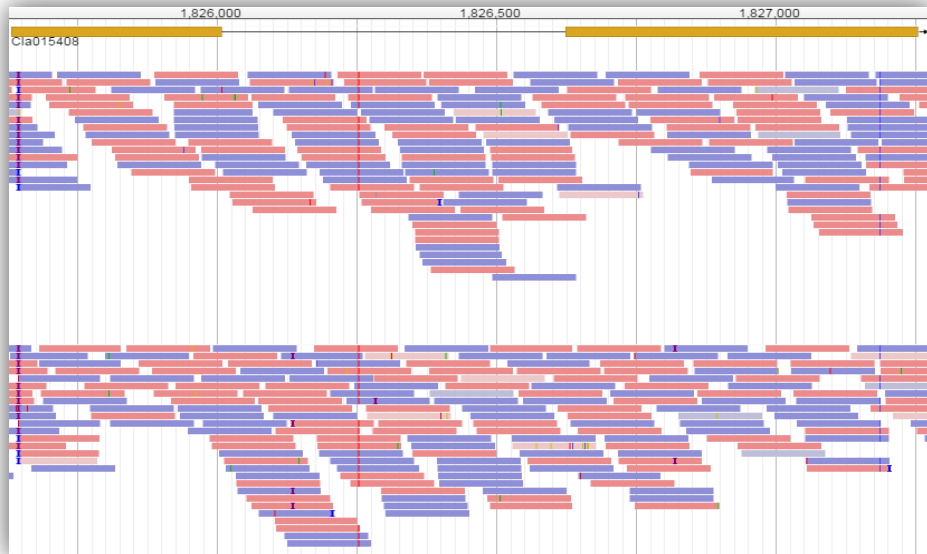

Cla015407

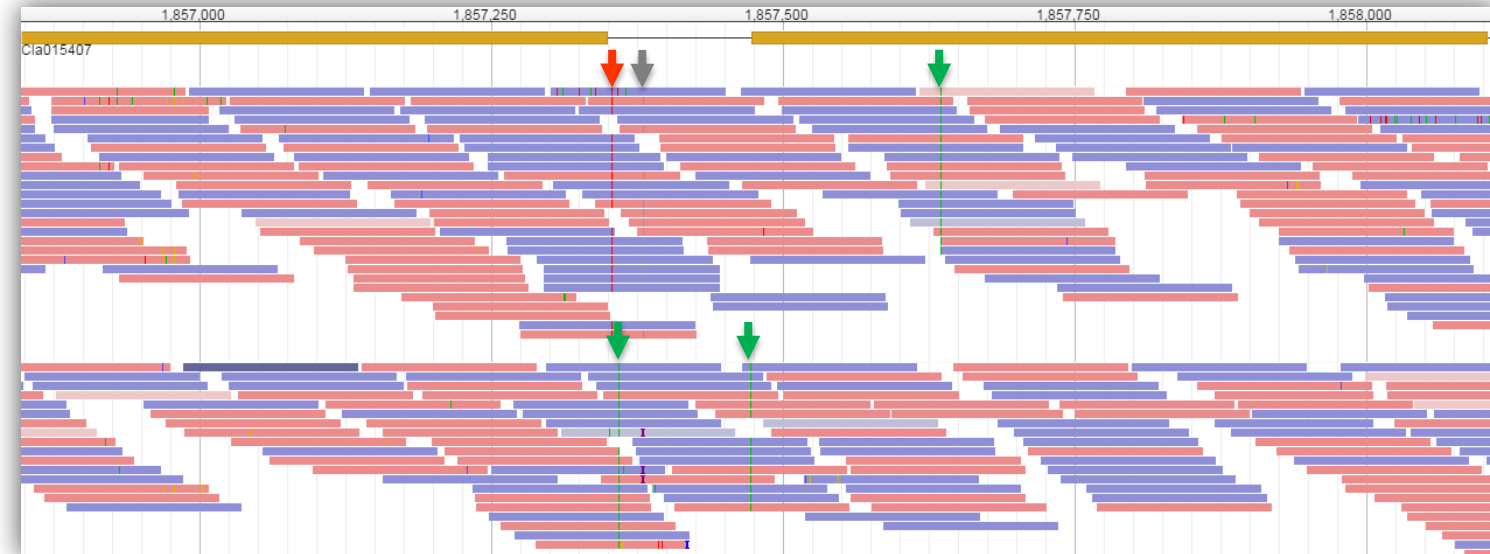

Cla015406

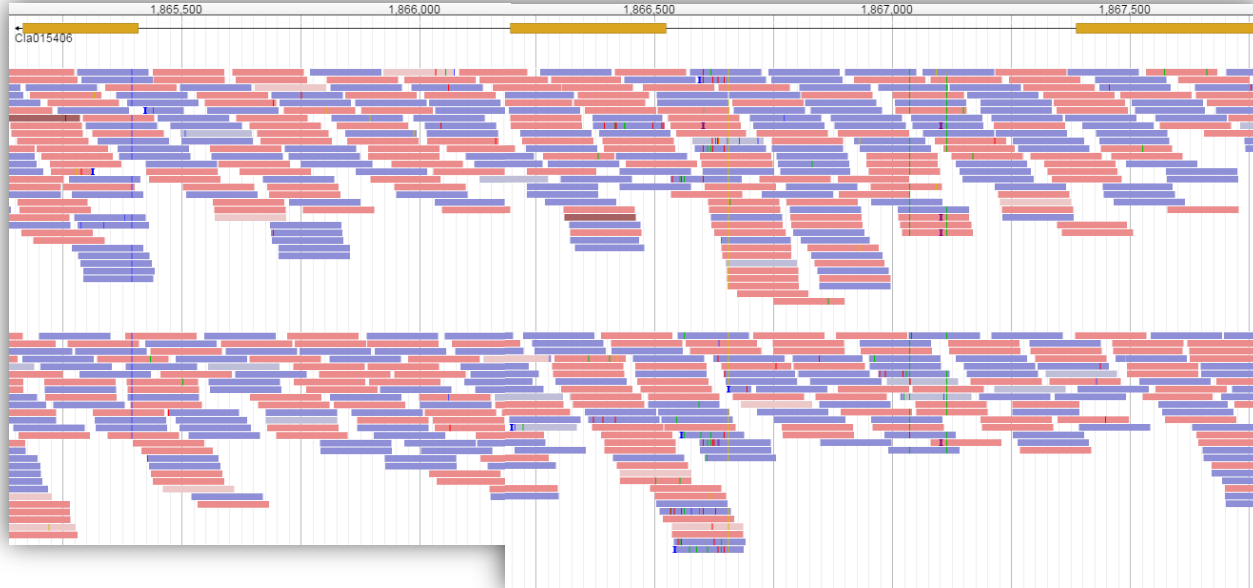

Cla015405

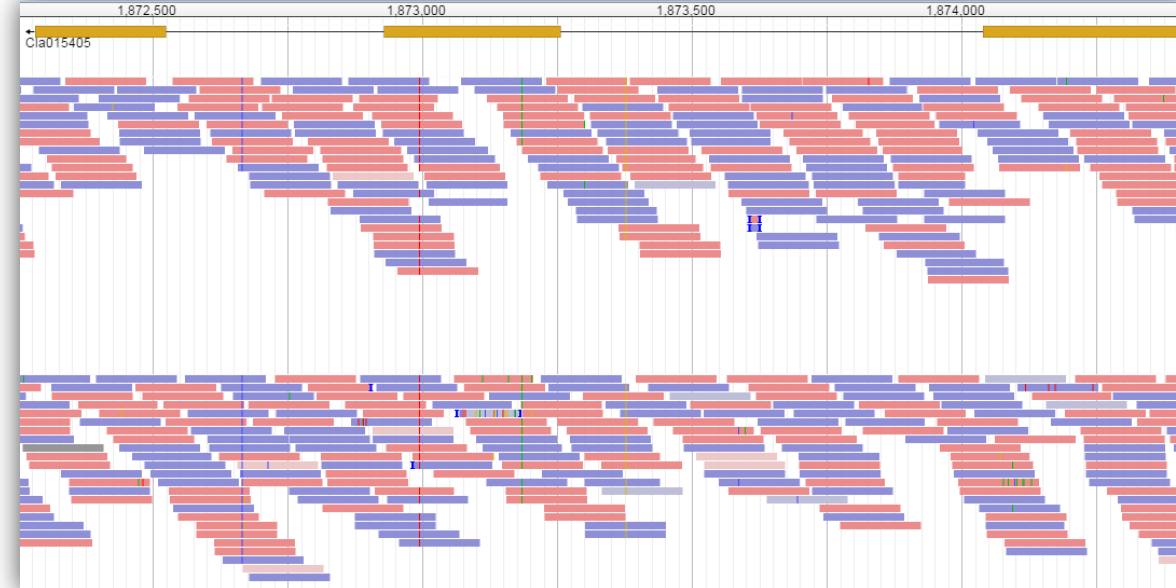

Supplement: Supplementary file 7 — Fig S2 [file 41438_2019_213_MOESM7_ESM.pdf]

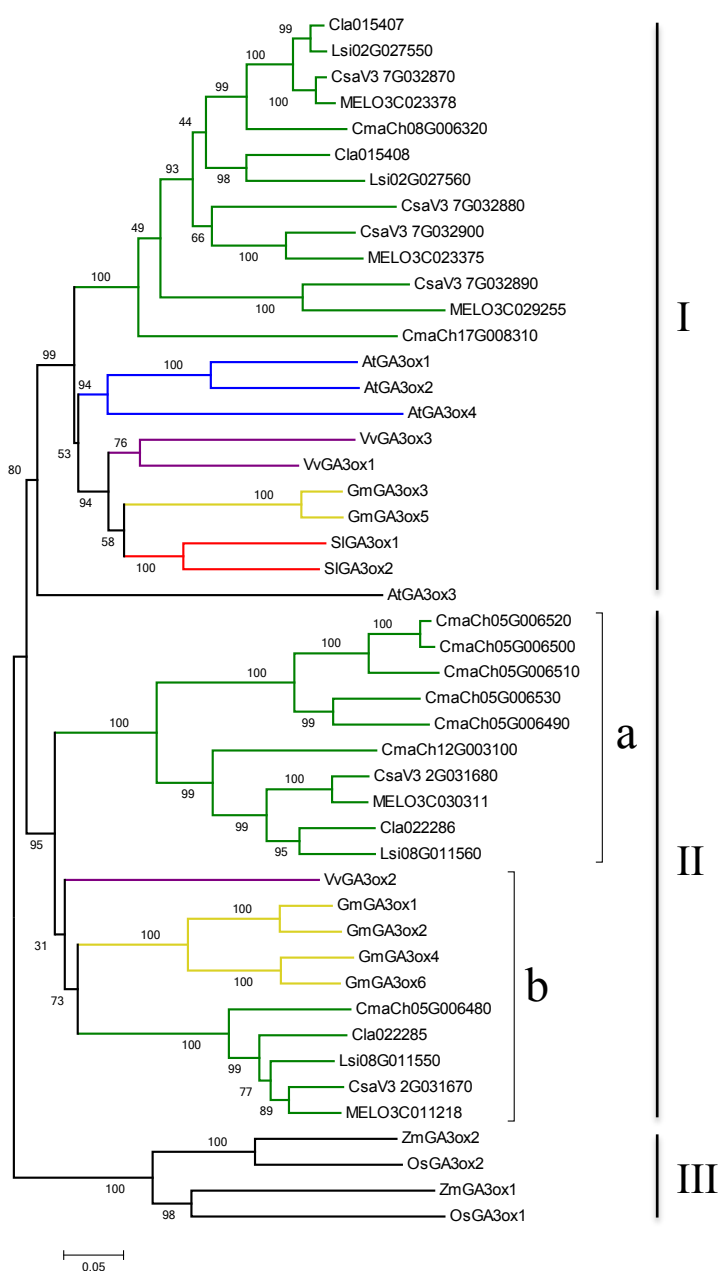

Supplement: Supplementary file 10 — Fig S5 [file 41438_2019_213_MOESM10_ESM.pdf]
