# Supplementary material for: A point mutation resulting in a 13 bp deletion in the coding sequence of Cldf leads to a GA-deficient dwarf phenotype in watermelon
Source: Hortic Res. 2019 Dec 1;6:132. doi: 10.1038/s41438-019-0213-8 (PMC6885051; doi:10.1038/s41438-019-0213-8)
Supplement: Supplementary file 8 — Fig S3 [file 41438_2019_213_MOESM8_ESM.pdf]

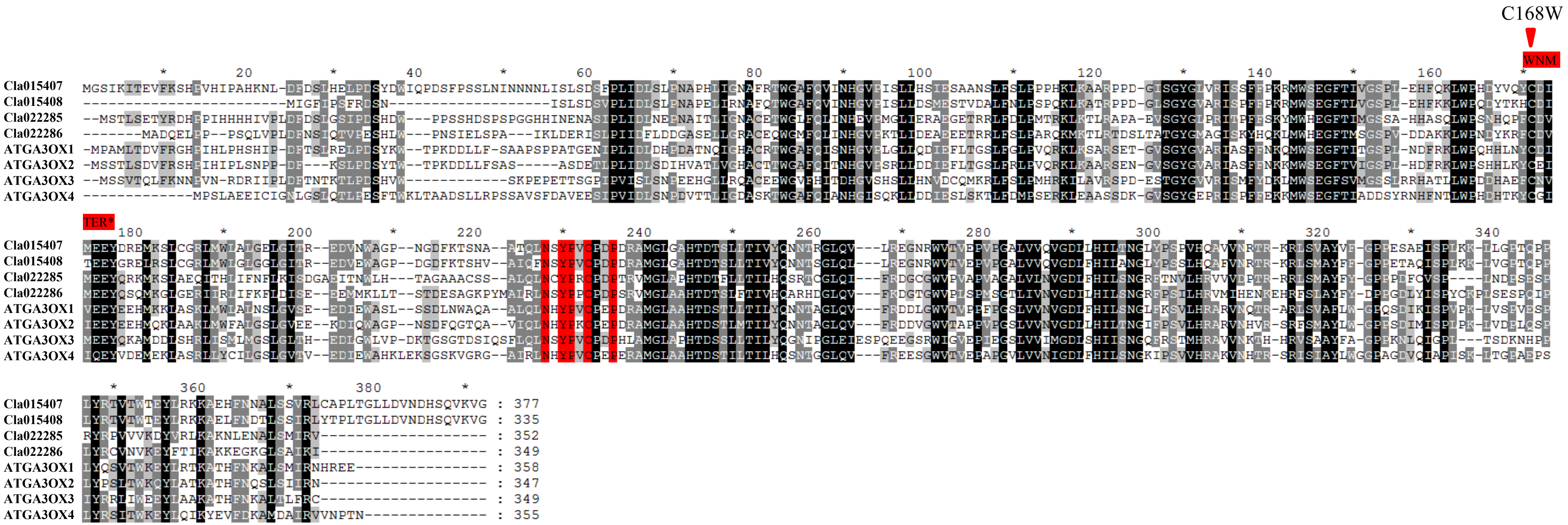
C168W
WNM
TER*
Cla015407
Cla015408
Cla022285
Cla022286
ATGA3OX1
ATGA3OX2
ATGA3OX3
ATGA3OX4
: 377
: 335
: 352
: 349
: 358
: 347
: 349
: 355
