# Supplementary material for: A point mutation resulting in a 13 bp deletion in the coding sequence of Cldf leads to a GA-deficient dwarf phenotype in watermelon
Source: Hortic Res. 2019 Dec 1;6:132. doi: 10.1038/s41438-019-0213-8 (PMC6885051; doi:10.1038/s41438-019-0213-8)
Supplement: Supplementary file 9 — Fig S4 [file 41438_2019_213_MOESM9_ESM.pdf]

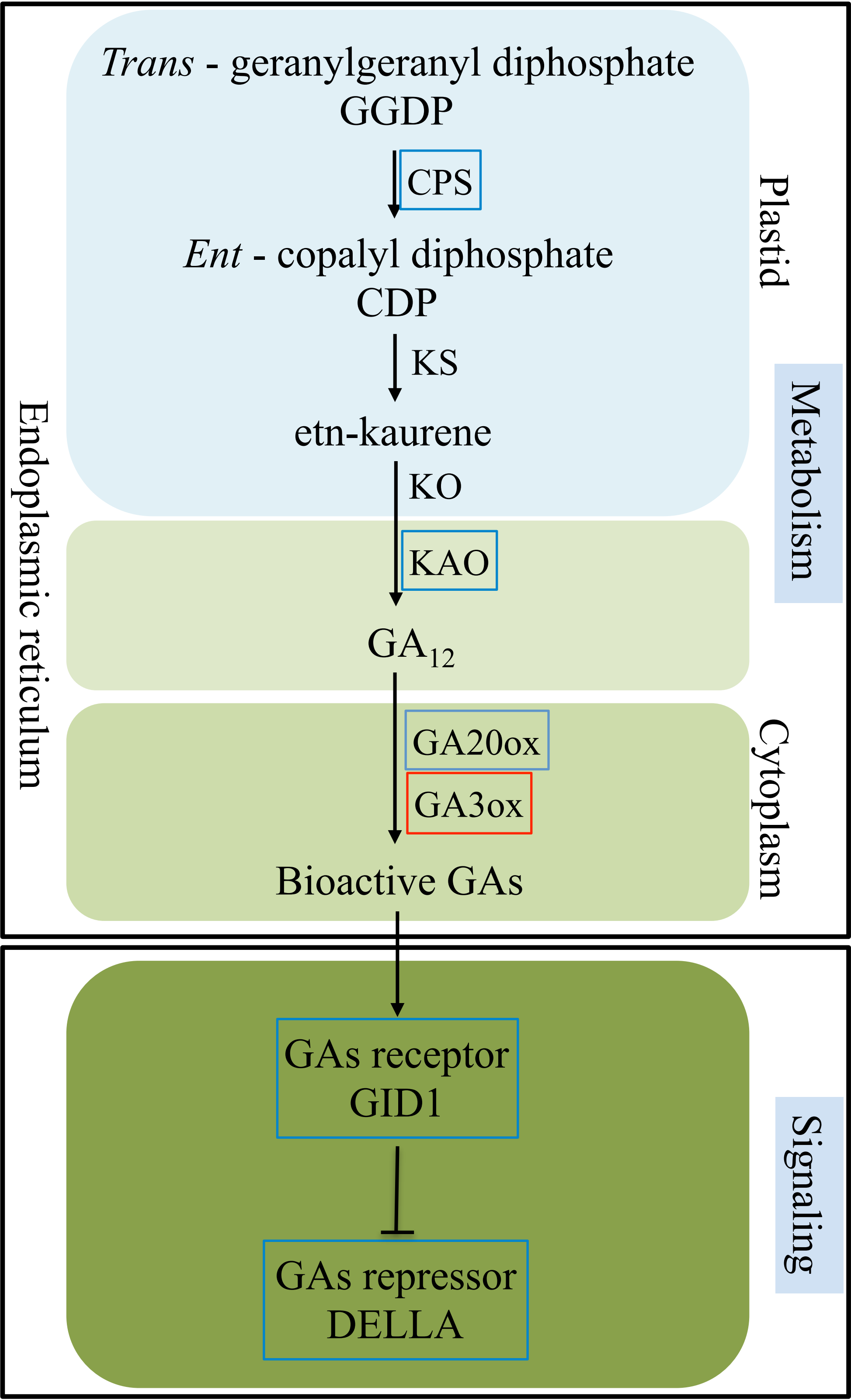
Trans - geranylgeranyl diphosphate
GGDP
CPS
Ent - copalyl diphosphate
CDP
KS
etn-kaurene
KO
KAO
GA_{12}
GA20ox
GA3ox
Bioactive GAs
GAs receptor
GID1
GAs repressor
DELLA
Plastid
Metabolism
Endoplasmic reticulum
Cytoplasm
Signaling
